# Immune Toxicity with Checkpoint Inhibition for Metastatic Melanoma: Case Series and Clinical Management

**DOI:** 10.1155/2018/9602540

**Published:** 2018-01-21

**Authors:** Anna J. Lomax, Jennifer Lim, Robert Cheng, Arianne Sweeting, Patricia Lowe, Neil McGill, Nicholas Shackel, Elizabeth L. Chua, Catriona McNeil

**Affiliations:** ^1^Chris O'Brien Lifehouse, Camperdown, NSW, Australia; ^2^Concord Repatriation General Hospital, Sydney, NSW, Australia; ^3^Centenary Institute, Sydney, NSW, Australia; ^4^Sydney Medical School, University of Sydney, Camperdown, NSW, Australia; ^5^Royal Prince Alfred Hospital, Camperdown, NSW, Australia

## Abstract

Immune checkpoint inhibitors (anti-PD-1 and anti-CTLA-4 antibodies) are a standard of care for advanced melanoma. Novel toxicities comprise immune-related adverse events (irAE). With increasing use, irAE require recognition, practical management strategies, and multidisciplinary care. We retrospectively evaluated the incidence, kinetics, and management of irAE in 41 patients receiving anti-PD-1 antibody therapy (pembrolizumab) for advanced melanoma. 63% received prior anti-CTLA-4 antibody therapy (ipilimumab). IrAE occurred in 54%, most commonly dermatological (24%), rheumatological (22%), and thyroid dysfunction (12%). Thyroiditis was characterised by a brief asymptomatic hyperthyroid phase followed by a symptomatic hypothyroid phase requiring thyroxine replacement. Transplant rejection doses of methylprednisolone were necessary to manage refractory hepatotoxicity. A bullous pemphigoid-like skin reaction with refractory pruritus responded to corticosteroids and neuropathic analgesia. Disabling grade 3-4 oligoarthritis required sulfasalazine therapy in combination with steroids. The median interval between the last dose of anti-CTLA-4 antibody and the first dose of anti-PD-1 therapy was 2.0 months (range: 0.4 to 22.4). Toxicities may occur late; this requires vigilance and multidisciplinary management which may allow effective anticancer therapy to continue. Management algorithms for thyroiditis, hypophysitis, arthralgia/arthritis, colitis, steroid-refractory hepatitis, and skin toxicity are discussed.

## 1. Introduction

Immune checkpoint inhibition is the established immunotherapy treatment for advanced melanoma. Induction of a tumour-directed immune response due to T-cell activation halts tumour evasion from immune surveillance [1, 2]. Blockade of cytotoxic T-lymphocyte antigen-4 (CTLA-4) with ipilimumab showed the first evidence of improved survival in advanced melanoma [3] and long-term survival can be achieved [3, 4]. Novel side-effects include autoimmune toxicities referred to as immune-related adverse events (irAE). With the increasing use of these agents (as monotherapy or in combination) irAE require recognition and practical management strategies.

Pembrolizumab and nivolumab are anti-programmed cell death 1 (PD-1) antibodies targeting the effector arm of the immune checkpoint pathway. Benefit has been demonstrated in ipilimumab pretreated and naïve patients [5]. Anti-PD-1 antibodies have superseded ipilimumab as a first-line immunotherapy treatment for advanced melanoma. Both anti-PD-1 agents have superior response rates (36–44%) [6, 7] compared to ipilimumab (13–19%) [6, 7] and improved 3-year survival (40–52%) [7, 8] versus 20–34% [3, 7]. Estimates of anti-PD-1 efficacy outside of clinical trials have been reported with response rates of 14–39% [9–11]. Anti-PD-1 agents have activity in other solid cancers including non-small cell lung cancer, genitourinary cancers, and Hodgkin's lymphoma [12–15].

Combining anti-PD-1 and anti-CTLA-4 checkpoint inhibitors improves response rate (58–61%) but at the cost of increased toxicity [7, 16]. Grade 3-4 or 3–5 treatment-related adverse events for combination therapy and anti-PD-1 or anti-CTLA-4 monotherapies have been reported in randomised trials: 45–59%, 17–21%, and 20–28%, respectively [6, 7, 16]. 3-year overall survival with dual checkpoint inhibition (nivolumab plus ipilimumab) is also superior to ipilimumab alone (58% vs 20–34%) [7] but what is critical is whether this adds a survival benefit over anti-PD-1 therapy alone given the added toxicity with this regimen. IrAE due to CTLA-4 blockade have an earlier onset and are more commonly associated with diarrhoea, colitis, and hypophysitis. Fatigue, arthralgia, and thyroid irAE are more frequently seen with PD-1 blockade [17]. IrAE with combination checkpoint inhibition can have a rapid onset and be associated with a protracted duration [18].

In clinical practice, patients are older with poorer Eastern Cooperative Oncology Group (ECOG) performance status than those enrolled in clinical trials. An early study of pembrolizumab after prior ipilimumab therapy required patients to have received the final dose of ipilimumab ≥ 6 weeks before commencing pembrolizumab [5], a period not necessarily pragmatic in clinical practice. Reduced dosing intervals between therapeutic agents impact severity and pattern of toxicities as observed in patients receiving these checkpoint inhibitors, albeit in different sequence [19].

This is a retrospective review of patients with advanced melanoma that received pembrolizumab at Chris O'Brien Lifehouse through compassionate access. We evaluated patients who were ipilimumab naïve and pretreated with respect to irAE and describe the management of these irAE in real clinical practice.

## 2. Methods

Patients with advanced melanoma were included. In patients who had received prior ipilimumab, disease had to be documented as progressive, recurrent, or persistent. Patients were excluded if they were receiving or were eligible for treatment with a BRAF or MEK inhibitor. Patients were also excluded if they had significant autoimmune disease requiring chronic immunosuppression.

Pembrolizumab was administered intravenously at 2 mg/kg of body weight every three weeks. Drug supply was via a compassionate access program. At the time of patient enrolment into the program, pembrolizumab therapy was not yet approved for use in Australia on the Pharmaceutical Benefits Scheme. Therapy was continued until disease progression or unacceptable toxicity. Response to pembrolizumab was assessed at week 12 after commencement and 12 weekly thereafter or as clinically appropriate. Where available, imaging was assessed according to Response Evaluation Criteria in Solid Tumours (RECIST) version 1.1. IrAE were graded according to the National Cancer Institute Common Terminology Criteria for Adverse Events, version 4.0 (CTCAE). Ethics committee approval was obtained (X15-0193 LNR/15/RPAH/256).

## 3. Results

From November 2013 to August 2015, 41 patients were identified. Patient and disease characteristics are described (Table 1). Median age was 65, 81% had an ECOG performance status of 0 or 1 and 71% had an elevated LDH. BRAF V600 mutations were identified in 24% of patients and 76% had M1c disease. Twenty-six patients (63%) had received prior ipilimumab. The median interval between the last ipilimumab dose and the first pembrolizumab dose was 2.0 months (range: 0.4 to 22.4). The median duration of follow-up was 4.1 months (range: 0 to 14.9). The median and mean number of pembrolizumab cycles received were 4 and 6, respectively (range: 1 to 20). In 15 patients, treatment was ongoing.

In patients whose tumour harboured a BRAF V600 mutation, one patient received one dose of pembrolizumab on the compassionate program while awaiting results of BRAF molecular testing. Once it was known that his tumour harboured an actionable V600K mutation, he was transitioned to dabrafenib plus trametinib and achieved a partial response. A second patient received 2 doses of pembrolizumab before also confirming the presence of a V600K mutation. This patient was commenced on dabrafenib and trametinib but developed progressive disease. Initial testing had demonstrated a negative immunostain for BRAF VE1 for these 2 patients but due to symptomatic disease needing swift commencement of treatment, pembrolizumab was started while formal molecular testing was performed.

The remaining 8 patients with a BRAF V600 mutation had received prior BRAF/MEK inhibitor therapy. These numbers are small and make it difficult to draw conclusions regarding response in this subgroup.

Objective response rates (ORR) were 26% and disease control rates (DCR) were 49% (*n* = 39, excluding the *n* = 2 BRAF V600K patients that received pembrolizumab before formal molecular results were reported). This was determined by RECIST 1.1 assessment for *n* = 20; imaging assessment but not per RECIST 1.1 *n* = 12 (imaging modality was not uniform between serial scans or performed offsite, precluding formal RECIST 1.1 assessment); clinical progression in *n* = 3 and unknown *n* = 4 patients. The median time to response was 2.7 months (range: 0.9 to 4.9). Three (7%) patients achieved a complete response.

IrAE were documented in 22 (54%) patients while receiving anti-PD-1 therapy. Common irAE were dermatological (24%), rheumatological (22%), and thyroid dysfunction (12%) (Table 1). Grade 3-4 irAE were uncommon (15%) with individual event rates of 2–5%. Of the 26 patients that received prior ipilimumab, 13 patients (50%) developed irAE secondary to ipilimumab. Of these 13 patients, 10 (38%) experienced subsequent irAE while receiving pembrolizumab. Of these 10 patients, 8 had an event of grade 1-2 severity during treatment with pembrolizumab and 2 developed grade 3-4 hepatotoxicity in the context of a short interval between ceasing ipilimumab and commencing pembrolizumab. Of the 13 patients who did not have irAE on ipilimumab, 6 (23%) patients had subsequent irAE while receiving pembrolizumab.

**Figure 1:**
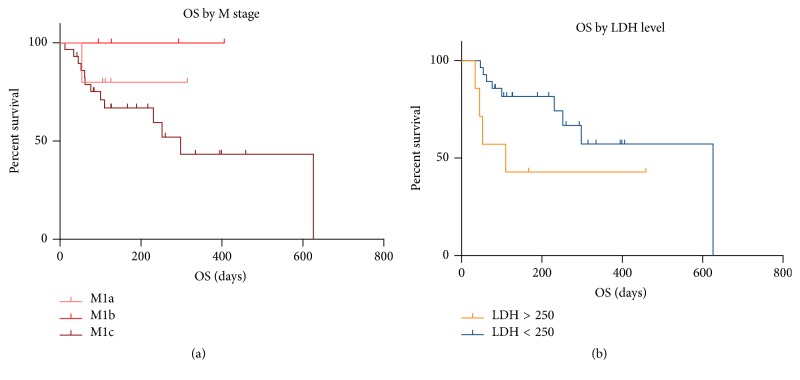
OS by M stage and LDH. Logrank statistic M stage (*p* = 0.28) and LDH level (*p* = 0.06).

Patients with M1c disease or an elevated LDH appeared to have worse survival outcomes (Figures 1(a) and 1(b)).

## 4. Organ Specific Toxicities and Treatment Algorithms

Owing to the mechanism of action of immunotherapy agents, manifestations of autoimmune toxicity can involve any organ. General principles of management revolve around managing mild (grade 1) toxicity with supportive measures and considering steroids in moderate (grade 2) toxicity. For severe (grades 3-4) toxicity, intervening with high-dose steroids or additional immunosuppressive therapy may be necessary. We outline management algorithms for the toxicities observed in our cohort of patients which overlap with established algorithms such as “eviQ Cancer Treatments Online” [20] but also include recommendations from our institutional experience. Specifically, we have documented general skincare supportive measures and the possible use of neuropathic analgesia for refractory pruritus. For endocrine toxicities, we have highlighted the expected clinical course of thyroiditis and have suggested a management algorithm for rheumatological irAE. Other guidelines, such as the ESMO Clinical Practice Guidelines [18], differ from the eviQ and our guidelines with respect to the grading of liver toxicity for autoimmune hepatitis. The ESMO statement refers to grade 3 ALT/AST elevation as 5–20x the upper limit of normal (ULN) and grade 4 elevation as >20x the ULN [18], whereas our algorithm and eviQ specify grade 3-4 AST/ALT elevation as >5x the ULN [20].

### 4.1. Dermatological Toxicity

Cutaneous toxicity of any grade occurred in 10 (24%) patients (Table 1). Common presentations included pruritus, cutaneous eruptions, for example, maculopapular, eczematous, flare of Grover's disease (transient acantholytic dermatosis), and less commonly vitiligo. Nine patients had rash/pruritus (one case involved a bullous pemphigoid-like reaction described below) and 1 patient was documented to have developed vitiligo. No presentations had mucosal involvement.

**Figure 2:**
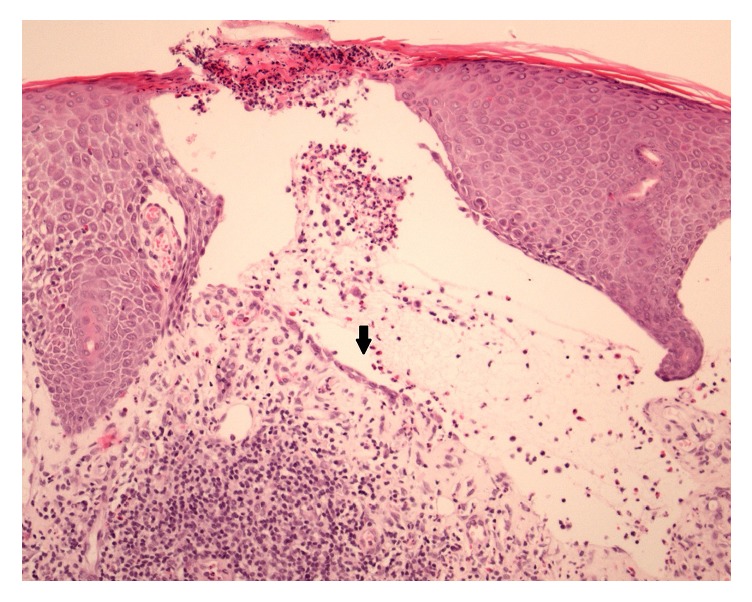
Pemphigoid-like reaction. Skin biopsies demonstrated a subepidermal blister (arrow) with inflammatory cells, predominantly eosinophils.

An elderly female developed grade 3 skin toxicity on pembrolizumab [21] having displayed grade 1 toxicity (pruritic maculopapular eruption) during prior ipilimumab therapy. The latter improved with supportive therapy. Due to progressive metastatic disease, pembrolizumab was commenced 6 weeks after ipilimumab cessation. A bullous pemphigoid-like drug reaction developed after 8 months of pembrolizumab. Skin biopsies demonstrated a subepidermal blister with inflammatory cells, predominantly eosinophils. A perivascular and interstitial inflammatory cell infiltrate of lymphocytes, eosinophils, and neutrophils was within the dermis with adjacent spongiotic epidermis (Figure 2). Direct immunofluorescence was negative. Pembrolizumab was ceased. Although the rash was steroid responsive, the pruritus proved refractory until pregabalin was commenced (25 mg daily and titrated to 25 mg twice daily). Neuropathic analgesia [22, 23] has shown efficacy in the management of uraemic pruritus; however, oversedation was observed in 12–30% [23]. The patient had a complete disease response to pembrolizumab.

### 4.2. Dermatological irAE Management Algorithm

Rash due to anti-PD-1 antibodies may occur in 13–26% and 15–33% for anti-CTLA-4 therapy. Pruritus can occur in 14–19% and 25–35%, respectively [17, 24]. Skin toxicity due to anti-PD-1 therapy is potentially mediated by a shared antigen coexpressed by the dermoepidermal junction and tumour cells [25]. Most presentations are mild, usually a nonspecific maculopapular pruritic eruption, occasionally eczematous. As would be expected in immunotherapy of melanoma, hypopigmentation and depigmentation (vitiligo) have been reported [26, 27] with sites of predilection, including trunk and limbs, but can be limited to photoexposed sites. Rarely, life-threatening conditions such as Stevens-Johnson syndrome (SJS) or toxic epidermal necrolysis (TEN) occur [28–30].

Upon development of a nonspecific rash, flare of preexisting dermatosis and secondary cutaneous infections needs to be excluded. Perform skin swabs (bacterial MCS, viral PCR) and scrapings (fungal KOH) if indicated. Skin biopsy (with direct immunofluorescence if blisters present) and FBC may reveal eosinophilic infiltrate and eosinophilia, respectively, supporting the diagnosis of drug exanthem. Tissue reactions are most commonly spongiotic or lichenoid in nature.

The majority of skin irAE are managed supportively. Avoidance of common irritants (soap and excess water) should be reinforced. Liberal amounts of emollients and ointment-based preparations, if xerotic or pruritic, are advised. Specific therapies include moderate to very potent topical corticosteroid ointments (under wet dressing occlusion) and low to high doses of oral antihistamines. Severe skin toxicity requires hospital admission and high doses of corticosteroid administration [28–30] orally or intravenously. Consider commencement of IVIG and/or cyclosporine if SJS/TEN is suspected or confirmed on urgent skin biopsy. Transfer to a burns unit is essential if skin loss is >10%. The management of skin toxicity is outlined in Table 2.

### 4.3. Gastrointestinal and Hepatic Toxicity

Gastrointestinal irAE were infrequent, occurring in 3 (7%) patients, with none experiencing grade 3 or 4 toxicity (Table 1). One patient developed grade 2 colitis with proctitis and had never received prior ipilimumab. Two ipilimumab pretreated patients developed diarrhoea of grade 1-2 severity.

Two patients (5%) experienced grade 3-4 hepatotoxicity (Table 1). A 58-year-old male received 4 cycles of ipilimumab with the final dose administered 22 days before starting pembrolizumab. Liver function test derangement (LFT) of grade 4 severity with transaminases 15–30 times the ULN and elevated bilirubin occurred one month later. Biopsy confirmed lobular and portal hepatitis consistent with drug-induced injury. Methylprednisolone 0.5 mg/kg for 3 days was initiated and escalated to 1 g for 3 days with concurrent mycophenolate mofetil 500 mg twice daily due to lack of improvement. Prednisolone was weaned and mycophenolate mofetil continued with resolution of biochemical abnormalities. Due to significant immunosuppression he developed presumed pneumocystis jiroveci pneumonia and died of respiratory failure and sepsis.

The second patient was a 67-year-old male who had a single dose of ipilimumab 27 days prior to commencing pembrolizumab. Following the second cycle of pembrolizumab he presented with fever and grade 3 ALT elevation at >5 times the ULN. Liver biopsy showed nonspecific mild portal and interface hepatitis. Methylprednisolone 1 mg/kg was commenced followed by weaning prednisolone and mycophenolate mofetil 1 g twice daily. The LFT derangement resolved. Due to this irAE and progressive disease, he did not receive subsequent therapy and died 6 months later.

### 4.4. Management of Refractory Hepatitis and Gastrointestinal Toxicity

Gastrointestinal toxicity is more commonly described with ipilimumab. Any grade of diarrhoea may occur in 23–33% with 3–6% of patients experiencing severe diarrhoea. Rates for significant colitis are 7–9% [17, 24]. Lower rates are documented with anti-PD-1 therapy with grade 3-4 or 3–5 diarrhoea described in 1–3% and similarly for severe colitis [17, 24]. Rates of severe diarrhoea or colitis for combined therapy are 9% and 8%, respectively [17, 24]. Management of colitis due to ipilimumab is well documented [31]. The management of single agent and combination checkpoint inhibitor induced colitis are outlined in Table 3.

In clinical trials, the rate of significant hepatitis or deranged liver function is identified at <1-2% for anti-CTLA-4 agents and 1-2% with anti-PD-1 therapies but increased to 6–8% with combined therapy [17, 24]. Grade 3-4 hepatotoxicity in our cohort was 5% and may reflect a short dosing interval between checkpoint inhibitors. The 2 patients in our cohort who developed hepatic toxicity required treatment with mycophenolic acid. Mycophenolate mofetil exerts its immunosuppressive effects through a cytostatic effect on lymphocytes [32]. Its use is outlined in clinical trial and ipilimumab irAE guidelines [31]. Doses of methylprednisolone required to treat transplant rejection have been used for ipilimumab-induced hepatotoxicity at our institution [33] and were necessary in 1 patient from our study cohort. From our experience, methylprednisolone 15 mg/kg (maximum 1 gm/day) is suggested for steroid-refractory hepatitis and is considered part of our management algorithm at our institution (Table 3).

### 4.5. Endocrine Toxicity, Thyroiditis-Kinetics, and Management

**Figure 3:**
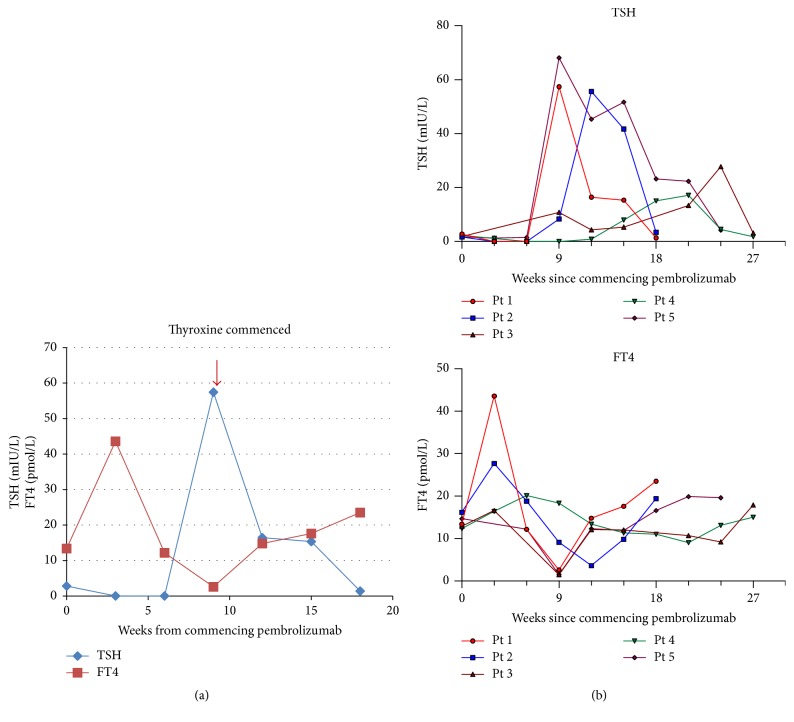
(a) Thyroiditis: hyperthyroid phase followed by hypothyroid phase in a patient treated with pembrolizumab. (b) Hyperthyroidism generally occurred within 3–6 weeks of initiation of pembrolizumab therapy (time = 0). Hypothyroid phase was evident by week 9 of pembrolizumab therapy and was characterised by markedly elevated TSH levels.

Thyroid dysfunction of grade 2 severity occurred in 5 (12%) patients (Table 1). Four patients had received prior ipilimumab. The onset of biochemical hyperthyroidism signalled the development of clinical thyroiditis in all patients. Thyroiditis was characterised by a brief asymptomatic hyperthyroid phase followed by a symptomatic hypothyroid phase (Figure 3(a)) requiring thyroxine replacement. This pattern is consistent with other case series [34, 35]. Hyperthyroidism occurred within 3–6 weeks of pembrolizumab initiation. The hypothyroid phase was generally evident within 3 weeks of the onset of thyroiditis. In most cases, the hypothyroid phase was evident by week 9 of pembrolizumab therapy and was characterised by markedly elevated TSH levels (Figure 3(b)). Despite age and comorbidities of the patients, initiation of high-dose thyroxine (i.e., 100 mcg daily) at the onset of hypothyroidism was required. Thyroxine should be uptitrated to achieve normalisation of TSH. No evidence of thyroid function recovery occurred in our series, demonstrating the need for serial TSH monitoring.

From clinical trial data, hypophysitis is more commonly associated with ipilimumab and thyroid toxicity with anti-PD-1 agents [17]. In our cohort of 26 patients that had been pretreated with ipilimumab, 2 patients developed hypophysitis prior to receiving pembrolizumab and in 2 patients this occurred during anti-PD-1 therapy. Thyroiditis and hypophysitis management is described in Table 4.

### 4.6. Rheumatological Toxicity

Rheumatological toxicity occurred in 9 (22%) of patients (Table 1) and most events were managed with low-dose prednisolone. One patient developed a grade 3-4 irAE. He received prior ipilimumab without complication. A disabling inflammatory arthropathy occurred 8 months after starting pembrolizumab. Synovial fluid aspirate showed an inflammatory infiltrate (white cell count 16.4 × 10^9^/L) and no crystals were seen on microscopy, with CRP 60 and ESR 103 reflecting systemic inflammation. Rheumatoid factor, cyclic citrullinated peptide, and antinuclear antibodies and human leukocyte antigen B27 were negative. Anti-PD-1 therapy was ceased. The arthropathy was refractory to prednisolone doses up to 75 mg daily and required the addition of sulfasalazine, and the dose increased from 500 mg daily to 1 g twice daily over 10 days and continued with a good clinical response. Eight months after pembrolizumab cessation, prednisolone was weaned to 5 mg daily and sulfasalazine continued at 1 g twice daily. This patient achieved a complete disease response to pembrolizumab.

A 64-year-old male with a history of childhood glomerulonephritis developed significant sicca symptoms (xerophthalmia, xerostomia, and significant parotid swelling) after 3 cycles of pembrolizumab. Antinuclear antibody was elevated with high titres of 1 : 640 consistent with new onset immune-mediated Sjogren syndrome. He was treated with prednisolone 25 mg daily and the symptoms resolved, allowing steroids to be weaned. A postulated mechanism is an aberrant T-cell activation that is not dissimilar to that induced by graft versus host disease [36].

### 4.7. Arthralgia and Arthritis Management Algorithm

Rheumatological toxicity in clinical trials is higher for anti-PD-1 (8–12%) than anti-CTLA-4 therapy (5-6%) [17, 24]. Severe toxicity is rare but has also been reported in 2 patients that received prolonged pembrolizumab therapy [37]. Sulfasalazine was used with benefit and may be of use in conditions refractory to corticosteroids or to expedite steroid tapering. A suggested algorithm for the management of arthralgia and arthritis is outlined (Table 5).

### 4.8. Pneumonitis

Pneumonitis was observed in 2 (5%) patients of grade 2 severity. One patient had a short interval (47 days) between anti-CTLA-4 and anti-PD-1 dosing. Pneumonitis rates due to checkpoint inhibitors are low in the published literature (<1–4%) across several cancer types [12, 13, 17]. Pneumonitis is important to consider in the differential diagnosis of new cough or dyspnoea; however, other causes such as infection, exacerbation of airways disease, cancer progression, or sarcoidosis should be considered. Assessment requires imaging with CT, respiratory review, and in some circumstances, bronchoscopy. Grade 2 and Grade 3-4 pneumonitis require high-dose steroids with slow taper and prophylaxis against opportunistic infections [38].

No patients in our cohort experienced neurological toxicities. Fulminant myocarditis has been documented in patients receiving dual checkpoint inhibitor therapy [39] and in a patient that received prior combination therapy and later received single agent anti-PD-1 therapy [7]. 

## 5. Conclusion

Our cohort of patients receiving anti-PD-1 therapy for advanced melanoma included patients with poorer prognostic features with higher rates of elevated LDH, M1c disease, and poorer ECOG [5, 17], in comparison with patients enrolled on clinical trials. In the subset of patients that had a prior ipilimumab irAE, 62% and 23% developed mild irAE or no irAE on pembrolizumab, respectively, demonstrating that the development of irAE to anti-CTLA-4 therapy did not preclude subsequent treatment with another checkpoint inhibitor.

Recommencing anti-PD-1 therapy may be considered for selected significant irAE, dependent upon perceived benefits and risks of immunotherapy rechallenge. Toxicities may occur late and require vigilance and multidisciplinary management. This may allow effective anticancer therapy to continue. Limitations of the cohort are small patient numbers and retrospective nature of the data. Furthermore, a significant number of patients received first-line anti-CTLA-4 therapy. The current evidence is for anti-PD-1 agents to be delivered first-line. However, the cases highlight a range of autoimmune toxicities and management in patients treated outside of a clinical trial. The use of overlapping anti-CTLA-4 and anti-PD-1 therapies demonstrates potentially added toxicity, having relevance in view of treatment approaches heading towards dual combinations of these and newer agents.

## Figures and Tables

**Table 1:** Patient characteristics and immune-related adverse events.

Characteristics	Compassionate pembrolizumab (*n* = 41)	
Median age (range), yr	65 (37–90)	
Male sex, number (%)	32 (78%)	
Primary type		
Cutaneous	31 (76%)	
Mucosal	2 (5%)	
Occult	7 (17%)	
Unknown	1 (2%)	
ECOG performance status, number (%)		
0-1	33 (81%)	
>1	7 (17%)	
Unknown	1 (2%)	
Lactate dehydrogenase, number (%)		
≤ULN (≤250 U/L)	7 (17%)	
>ULN (>250 U/L)	29 (71%)	
Unknown	5 (12%)	
Metastasis stage, number (%)		
In-transit disease	1 (2%)	
M1a	5 (12%)	
M1b	4 (10%)	
M1c	31 (76%)	
Number of organ sites of disease		
≤3	24 (58%)	
>3	15 (37%)	
Unknown	2 (5%)	
BRAF V600 mutation, number (%)	10 (24%)	
Prior lines of treatment, number (%)		
0	10 (24%)	
1	20 (49%)	
2-3	10 (24%)	
Unknown	1 (2%)	
Preexisting autoimmune condition	2 (5%)	
Ipilimumab pretreated, number (%)	26 (63%)	
Ipilimumab-related irAE, number (%)	13 (50%)	

Rates of irAE during anti-PD-1 therapy	Any grade	Grade 3 or 4

Any	22 (54%)	4 (15%)
Skin	10 (24%)	1 (2%)
Arthralgia/arthritis	9 (22%)	1 (2%)
Thyroid dysfunction	5 (12%)	0
Gastrointestinal^*∗*^	3 (7%)	0
Hypophysitis	2 (5%)	2 (5%)
Hepatitis	2 (5%)	2 (5%)
Pneumonitis	2 (5%)	0
Uveitis	1 (2%)	0
Parotitis	1 (2%)	0

^*∗*^Colitis and proctitis *n* = 1 and diarrhoea *n* = 2.

**Table 2:** Skin irAE: management algorithm.

Dermatology irAE	Investigations	Management
*Grade 1/mild*		
Rash < 10% body surface area (BSA), pruritus		Continue checkpoint inhibitor therapy *General skin care measures:* Avoid irritants: soap and excess water Emollients: creams and ointments Oral antihistamines: Nonsedating (daytime); sedating (nocte) Topical corticosteroids (moderate potency, ointment > cream vehicle) Phototherapy for pruritus: short course narrow band UVB, for example, 3x week for 4 weeks (relatively contraindicated with history of melanoma)

*Grade 2/moderate*		
Rash (10–30% BSA), pruritus	Skin swabs MCS, viral PCR, scrapings (fungal KOH) Prolonged symptoms (1-2 weeks): consider skin biopsy	Continue checkpoint inhibitor therapy Consider dermatology review General skin care measures and emollients as above Oral antihistamines (increased dosing may be required: 2–4x standard dose), depending on renal and liver function Topical corticosteroid (moderate to very potent, ointment > cream vehicle) Wet dressings (educate at outpatient dermatology treatment centre) *Prolonged symptoms:* Delay immunotherapy until resolving to ≤ Grade 1 Prednisolone 0.5–1 mg/kg/day with slow taper Consider hospital admission for wet dressings *Refractory pruritus:* Consider neuropathic analgesia, for example, pregabalin 25 mg daily and titrate to response

*Grade 3-4/severe/life-threatening*		
Rash (≥30% BSA), pruritus, blisters, ulceration	Skin biopsy (with direct immunofluorescence if blisters present)	Delay immunotherapy if Grade 3 until resolving to Grade ≤ 1 Cease immunotherapy if SJS/TEN (Grade 4) Urgent dermatology review and biopsy Prednisolone 1 mg/kg/day or pulse with methylprednisolone 1-2 mg/kg/day for 3 days^*∗*^Consideration of IVIG and/or cyclosporin Transfer to burns unit if skin loss > 10%

^*∗*^Switch to oral prednisolone 1 mg/kg/day with slow taper over 1 month or longer. PJP (e.g., bactrim DS 1/2 tablet daily) and GIT ulcer prophylaxis therapy when patients are on prolonged steroid taper. Monitor blood glucose.

**(a):** 

Gastrointestinal irAE	Investigations	Management
*Grade 1/mild*		
Diarrhoea (<4 stools/day over baseline)	Stool MCS	Continue checkpoint inhibitor monotherapy (If on dual checkpoint inhibitor therapy, patient will need careful consideration and monitoring closely) Antimotility agents, for example, loperamide Fluid replacement If prolonged symptoms, treat as Grade 2

*Grade 2/moderate*		
Diarrhoea (4–6 stools/day over baseline) Colitis (pain, mucus, or blood)	Stool MCS Consider colonoscopy	Delay immunotherapy until resolving to Grade ≤ 1 (If on dual checkpoint inhibitor therapy, consider ceasing anti-CTLA-4) Consider hospital admission Gastroenterology referral Oral prednisolone 1 mg/kg/day for colitis or persistent diarrhoea

*Grade 3-4/severe/life-threatening*		
Diarrhoea (≥7 stools/day over baseline, incontinence, life-threatening) Colitis (severe pain, blood, mucus, and peritonism)	Stools MCS Colonoscopy, if colitis suspected or persistent diarrhoea despite steroids AXR/CT if suspected perforation	Grade 3 toxicity: Delay anti-PD-1 until resolving to Grade ≤ 1 with careful consideration as to retreatment Cease anti-CTLA-4 Grade 4 toxicity (life-threatening, perforation): Discontinue immunotherapy permanently Hospital admission Gastroenterology referral Pulse with methylprednisolone 1-2 mg/kg/day^*∗*^ If no response to steroid therapy (3–5 days), consider infliximab 5 mg/kg (if no perforation/sepsis)

**(b):** 

Hepatic irAE	Investigations	Management
*Grade 1/mild*		
Hepatic (AST/ALT < 3x ULN and/or total bilirubin < 1.5x ULN)	LFTs and viral serology Monitor LFTs weekly Exclude disease progression or medication-related causes	Continue checkpoint inhibitor therapy

*Grade 2/moderate*		
Hepatic (AST/ALT >3–≤5x ULN and/or total bilirubin >1.5–≤3x ULN)	LFTs and viral serology Exclude disease progression or medication-related causes LFTs every 3 days	Delay checkpoint inhibitor therapy until improving to baseline Consider gastroenterology referral Consider oral prednisolone 1 mg/kg/day with slow taper

*Grade 3-4/severe/life-threatening*		
Hepatic (AST/ALT >5x ULN and/or total bilirubin >3x ULN)	LFTs and viral serology Exclude disease progression or medication-related causes LFTs daily	Discontinue checkpoint inhibitor therapy Hospital admission Gastroenterology referral Pulse with methylprednisolone 1-2 mg/kg/day for 3 days^*∗*^ *Steroid refractory hepatitis:* If no improvement after 3–5 days consider the following: Mycophenolate mofetil 500 mg–1 g bd and escalation of methylprednisolone to 15 mg/kg daily (maximum 1 gm/day) for 3 days

^*∗*^Switch to oral prednisolone 1 mg/kg/day with slow taper over 1 month or longer. PJP (e.g., bactrim DS 1/2 tablet daily) and GIT ulcer prophylaxis therapy when patients are on prolonged steroid taper. Monitor blood glucose.

**Table 4:** Endocrine irAE management algorithm.

Endocrine irAE	Investigations	Management
*Grade 1/mild*		
Thyroid dysfunction (asymptomatic)	TFTs (TSH, FT4, FT3)	Continue checkpoint inhibitor therapy Mild biochemical abnormality: monitor TFTs prior to each infusion Consider endocrine referral

*Grade 2/moderate*		
Thyroiditis (initial hyperthyroid phase preceding prolonged hypothyroid phase)	TFTs prior to each infusion	Continue checkpoint inhibitor therapy Endocrine referral Hyperthyroidism may require medical management, if symptoms exist, but with close monitoring as this phase is usually short-lived *Onset of hypothyroid phase (generally by week 9 of treatment):* Commence thyroxine 50–100 mcg/daily Increase by 50 mcg in 3 weeks if TSH is still high until TSH is within normal range Continue thyroxine maintenance dose
Hypophysitis (symptomatic but clinically stable)	(AM) ACTH, cortisol, TFTs, LH, FSH, testosterone, oestrogen, prolactin, GH, IGF-1, blood glucose MRI pituitary	Consider delay of checkpoint inhibitor therapy Prednisolone 1 mg/kg/day Taper glucocorticoid to maintenance oral hydrocortisone (e.g., 10 mg hysone 0600/1500) It will generally require lifelong physiological replacement of steroid Adrenal sick day education Commence thyroxine/gonadal hormone replacement if required^†^

*Grade 3-4/severe/life-threatening*		
Hypophysitis (adrenal crisis: fatigue, headache, dizziness, hypotension, and hypoglycaemia shock)	(AM) ACTH, cortisol, TFTs, LH, FSH, testosterone, oestrogen, prolactin, GH, IGF-1, blood glucose MRI pituitary	Delay checkpoint inhibitor therapy Urgent endocrine review Pulse with methylprednisolone 1-2 mg/kg/day if indicated (e.g., headache); or high dose intravenous glucocorticoids (i.e., hydrocortisone 50 mg QID) Manage/exclude sepsis Fluid replacement Taper glucocorticoid to maintenance oral hydrocortisone (e.g., 10 mg hysone 0600/1500) It will generally require lifelong physiological replacement of steroid Adrenal sick day education Commence thyroxine/gonadal hormone replacement if required^†^

^†^Ensure steroid repletion prior to initiation of thyroxine to avoid precipitating adrenal crisis. Gonadal hormone replacement therapy can be initiated nonurgently when hypogonadotropic hypogonadism (secondary to hypophysitis) is confirmed to be persistent.

**Table 5:** Suggested algorithm arthralgia and arthritis.

Rheumatological irAE	Investigations	Management
*Grade 1/mild*		
Arthralgia and arthritis (minimal symptoms or signs)		Continue checkpoint inhibitor therapy Simple analgesia as required

*Grade 2/moderate*		
Arthralgia and arthritis (moderate pain, inflammation, and impacting on daily function)	*Exclude:* Sepsis, crystal-induced arthritis, and coincidental inflammatory arthritis *Perform:* Synovial fluid cell count, polarised microscopy for crystals, gram stain and culture, blood culture RF, CCP, ANA, HLA-B27 (if positive, consider coincidental disease)	Consider delay of checkpoint inhibitor therapy Consider rheumatology referral *Monoarthritis or oligoarthritis:* Consider intra-articular corticosteroid *Moderate inflammatory arthritis:* Consider low dose prednisolone 5–10 mg daily For more significant symptoms, higher doses may be required, for example, prednisolone 25 mg daily If response is not rapid, consider addition of sulphasalazine (immunomodulator without immunosuppressive effect)

*Grade 3-4/severe/life-threatening*		
Arthralgia and arthritis (severe pain or inflammation, disabling, and impacting on self-care)	*Exclude:* Sepsis, crystal-induced arthritis, and coincidental inflammatory arthritis *Perform:* Synovial fluid cell count, polarised microscopy for crystals, gram stain and culture, blood culture RF, CCP, ANA, HLA-B27 (if positive, consider coincidental disease)	Discontinue immunotherapy Rheumatology referral *Moderate-severe inflammatory arthritis:* Prednisolone 25 mg–40 mg daily If response is not rapid, consider addition of sulphasalazine *Severe:* Pulse with methylprednisolone 1-2 mg/kg/day for 3 days^*∗*^

^*∗*^Switch to oral prednisolone 1 mg/kg/day with slow taper over 1 month or longer. PJP (e.g., bactrim DS 1/2 tablet daily) and GIT ulcer prophylaxis therapy when patients are on prolonged steroid taper. Monitor blood glucose.
